# Editorial: Systems Biology Methods in Computational Immuno-Oncology

**DOI:** 10.3389/fgene.2022.885252

**Published:** 2022-04-08

**Authors:** Andrei S. Rodin, Mohamed Uduman, Peter P. Lee, Francesco Maria Marincola, Sergio Branciamore

**Affiliations:** ^1^ Department of Computational and Quantitative Medicine, Beckman Research Institute, City of Hope National Medical Center, Duarte, CA, United States; ^2^ Cell Signaling Technology, Danvers, MA, United States; ^3^ Department of Immuno-Oncology, Beckman Research Institute, City of Hope National Medical Center, Duarte, CA, United States; ^4^ Kite Pharma, Santa Monica, CA, United States

**Keywords:** systems biology, computational biology, immuno-oncology, immunotherapy, immune scores, immune response, immune prognostic signature

Automated analysis, modeling, and interpretation of the high-dimensional multimodal data is an active research direction in the areas of immunology and immuno-oncology. In general, there is a growing need for the systems biology perspective and data analyses, as well as the corresponding computational methodology development, in these interrelated fields.

For this Research Topic, we sought both applied and computational/methodological research papers at the interface of immuno-oncology and computational systems biology, with an emphasis on immune scores and prognostic signatures.

Multi-modal, integrative, network analyses emerged as a common theme in the immuno-oncological computational systems biology domain. Zhao et al. presented an analysis strategy involving protein-protein-interaction (PPI) and TF-lncRNA-miRNA-mRNA regulatory networks to identify differentially expressed prognostic genes and scrutinize the potential regulatory mechanisms in cervical cancer tumor immune microenvironments. Four candidate prognostic genes were identified from the TCGA data and validated in the independent GEO cervical cancer cohort, with an eye towards increasing immunotherapy efficiency and exploration of novel treatment avenues. This study highlighted the utility of combining PPI and multifactor regulatory networks in a unified analysis framework; such multi-level network analyses (whether carried out *via* sequential rounds of network modeling, or by combining different modalities and data types in the same network construction) are a clear and welcome trend in the field.

Likewise, Chen et al. concentrated on cervical cancer but used LASSO regression to construct and refine the immune prognostic signature based on the lncRNA data. The resulting six-lncRNA immune prognostic signature (LIPS) was *in silico* validated by training/testing dataset subdivision, and its prognostic accuracy was estimated by ROC curves, univariate, and multivariate Cox regression analyses, using different datasets. This compact LIPS was shown to be an independent risk factor for cervical cancer, thus adding to both more accurate prognostic assessment and, potentially, further insights into immunotherapy strategies. While no network modeling *per se* was performed, gene set enrichment analysis suggested LIPS association with the immune response and tumor-related signaling pathways; subsequent scrutiny revealed patients with high and low LIPS being in different immune states.

Moving on from cervical cancer to lung adenocarcinoma, Zhu et al. also adopted an integrative network approach to identifying and validating immune signatures. Here, TCGA and GEO databases were screened for differentially expressed genes, and a PPI network was constructed for enriched immune-related genes. The prognostic role of “hub” genes in the network was assessed *via* the Kaplan-Meyer plotter and OncoLnc databases; qualified “hub” immune-related genes subsequently led to the lncRNA–miRNA–mRNA ceRNA (competing endogenous RNA) network construction. Tumor-infiltrating immune cells’ landscape was also explored, and linked to the “hub” genes. Ultimately, two immune-related ceRNA networks were found to be involved in lung adenocarcinoma progression, presenting two novel directions for targeted immunotherapy.

Network modeling on the cell level, namely patient-specific cell-cell communication, was the subject of Gibbs et al. study, wherein high-level network features, representing signaling “axes”, were linked with the clinical outcomes from the large-scale TCGA patient samples’ datasets. Cell-cell edges associated with predominant (for individual tumor types) cell subtypes were found to be prognostic and predictive with respect to clinical phenotypes; in addition, genes from the differential networks were used for gene ontology term enrichment, leading to, among others, five immune-related themes. As a part of the study, novel methodologies for cell-cell communication network construction, weighting, and interpretation were developed and tested.

The above studies exemplify the emerging and prospective trends in immuno-oncological computational systems biology research: first, integrated and multi-level network analyses; second, emphasis on the wide variety of methods, ranging from “conventional” statistical and machine learning approaches to novel and original network-centered algorithms and tools; third, combining different available modalities into the single analysis framework/pipeline. The two major challenges facing the field are the robustness of the network modeling, and the sheer variety of the methods and their assumptions. These two issues are interrelated. For example, there is only a limited concordance between the different network modeling methods in the scRNA-seq gene regulatory network space, and many of the network modeling methods are unsuccessful in recovering the whole “ground truth” (Chen and Mar 2018; Pratapa et al., 2020). Likewise, “conventional” immuno-oncology data analysis methods might miss strong signals due to the violation of linearity, normality, and other assumptions (Rodin et al., 2021). We believe that the solution lies in 1) development and application of the variety of methods, with different explicit and implicit assumptions, and 2) concentrating on, and highlighting, smaller sub-networks of interest—such networks can be more easily validated and refined *in silico*, thus partially sidestepping the “dimensionality curse”. The research papers in this Research Topic are exemplary in these regards, and clearly outline the attractive methodological template for ongoing and future investigations in the field.

It remains to note that while most of the so-called multi-level or integrative network analyses are in fact sequential (moving from one modality to another), it would be beneficial to incorporate many different immuno-oncology–related datatypes into the same multiscale network, similar to the recent methodological work in other domains, such as Alzheimer’s Disease -omics (Beckmann et al., 2020). There are methodological and computational difficulties involved, but they can be overcome, at least on the smaller sub-networks level, by 1) incorporating mixed-type metrics (such as mixed mutual information) and network scoring functions into the network-building algorithms, and 2) appropriately harmonizing the resulting key network features and corresponding signals. This is a promising research direction that will lead to an even more integrative, systems-level, analysis and interpretation of the immuno-oncology data.
